# Reply to Itagaki et al. Comment on “Retuerto et al. Analysis of Gut Bacterial and Fungal Microbiota in Children with Autism Spectrum Disorder and Their Non-Autistic Siblings. *Nutrients* 2024, *16*, 3004”

**DOI:** 10.3390/nu17233682

**Published:** 2025-11-25

**Authors:** Mauricio Retuerto, Hilmi Al-Shakhshir, Janet Herrada, Thomas S. McCormick, Mahmoud A. Ghannoum

**Affiliations:** 1 Department of Dermatology, Case Western Reserve University, Cleveland, OH 44106, USA; 2 Center for Medical Mycology, University Hospitals Cleveland Medical Center, Cleveland, OH 44106, USA

Thank you for your commentary on our recent article, “Analysis of Gut Bacterial and Fungal Microbiota in Children with Autism Spectrum Disorder and Their Non-Autistic Siblings” (Retuerto et al., 2024) [1]. We appreciate your viewpoint and the opportunity to address your concerns.

## 1. Principal Component Analysis (PCA) and Principal Coordinate Analysis (PCoA)

Your point regarding the simultaneous use of PCA and PCoA is not accurate. PCA and PCoA can, and often are, performed simultaneously. In fact, microbiome-related R packages offer these methods simultaneously. Although PCA and PCoA are both methods to reduce data dimensionality, they serve slightly different purposes, based on different mathematical foundations. PCA is generally used for continuous data and relies on Euclidean distances, while PCoA can be applied to any distance or dissimilarity matrix, making it more versatile for datasets such as microbiomes.

In our study, we employed both methods to provide complementary insights into the microbiome community. PCA was used to highlight variance within the dataset, while PCoA was utilized to capture the distances between samples. This dual approach is common in microbiome research to ensure robustness and comprehensiveness of the findings, as demonstrated by two recent publications (one in the autism space) using the exact same approach [2,3]. As a point of clarity, we would like to confirm that the identified bacterial and fungal datasets were analyzed separately, not together.

## 2. Use of Non-Parametric Tests

Regarding the use of non-parametric Spearman correlation and Wilcoxon rank-sum tests, we agree that these methods are typically applied to data that do not assume a normal distribution. However, the standardization of operational taxonomic units (OTUs) does not preclude the use of non-parametric tests. Indeed, non-parametric methods can be particularly valuable in microbiome studies due to the general non-normal distribution of microbial abundance data.

## 3. Relative vs. Absolute Abundance

We agree that the distinction between relative and absolute abundance is important. In our study, we focused on relative abundance due to the inherent nature of 16S rRNA gene sequencing data, which provides proportional data rather than absolute counts. While absolute abundance data would certainly offer additional insights, it requires techniques such as quantitative PCR, which was not performed in the current study. However, relative abundance helps normalize the difference in total counts to begin with, allowing for better comparisons in relative composition.

## 4. Compositional Data Analysis

Your recommendation to use centered log-ratio (CLR) or additive log-ratio (ALR) transformations, as you have pointed out in other peer-reviewed [4] and non-peer-reviewed reports [5], is an appreciated additional perspective. These transformations address the compositional nature of microbiome data and may refine issues related to relative abundance. We appreciate this suggestion and will consider incorporating it in future analyses.

## Data Availability

All data generated or analyzed during this study are included in this published article. The datasets generated during and/or analyzed during the current study are available from the corresponding author on reasonable request.
